# Vectorial competence, insecticide resistance in *Anopheles funestus* and operational implications for malaria vector control strategies in Benin Republic

**DOI:** 10.1186/s12936-023-04815-9

**Published:** 2023-12-21

**Authors:** Romaric Akoton, Pierre Marie Sovegnon, Oswald Y. Djihinto, Adandé A. Medjigbodo, Romuald Agonhossou, Helga M. Saizonou, Genevieve M. Tchigossou, Seun M. Atoyebi, Eric Tossou, Francis Zeukeng, Hamirath O. Lagnika, Wassiyath Mousse, Ayola Akim Adegnika, Rousseau Djouaka, Luc S. Djogbénou

**Affiliations:** 1 Tropical Infectious Diseases Research Centre (TIDRC), University of Abomey-Calavi, Abomey-Calavi, Benin; 2 Fondation Pour la Recherche Scientifique (FORS), Cotonou, Benin; 3 International Institute of Tropical Agriculture, Cotonou, Benin; 4 Cell Biology and Genetics Unit, Department of Zoology, University of Ibadan, Ibadan, Oyo Nigeria; 5 Department of Biochemistry and Molecular Biology, Faculty of Science, University of Buea, Buea, Cameroon; 6 Centre de Recherches Médicales de Lambaréné (CERMEL), Lambaréné, Gabon; 7 Institute for Tropical Medicine (ITM), University of Tübingen, Tübingen, Germany; 8 Regional Institute of Public Health, University of Abomey-Calavi, Ouidah, Benin

**Keywords:** *Anopheles funestus*, Insecticide resistance, Vectorial competence, Malaria, Benin

## Abstract

The primary reason for the failure of malaria vector control across endemic regions is the widespread insecticide resistance observed in *Anopheles* vectors. The most dominant African vectors of malaria parasites are *Anopheles gambiae* and *Anopheles funestus* mosquitoes. These species often exhibit divergent behaviours and adaptive changes underscoring the importance of deploying active and effective measures in their control. Unlike *An. gambiae*, *An. funestus* mosquitoes are poorly studied in Benin Republic. However, recent reports indicated that *An. funestus* can adapt and colonize various ecological niches owing to its resistance against insecticides and adaptation to changing breeding habitats. Unfortunately, scientific investigations on the contribution of *An. funestus* to malaria transmission, their susceptibility to insecticide and resistance mechanism developed are currently insufficient for the design of better control strategies. In an attempt to gather valuable information on *An. funestus*, the present review examines the progress made on this malaria vector species in Benin Republic and highlights future research perspectives on insecticide resistance profiles and related mechanisms, as well as new potential control strategies against *An. funestus*. Literature analysis revealed that *An. funestus* is distributed all over the country, although present in low density compared to other dominant malaria vectors. Interestingly, *An. funestus* is being found in abundance during the dry seasons, suggesting an adaptation to desiccation. Among the *An. funestus* group, only *An. funestus* sensu stricto (s.s.) and *Anopheles leesoni* were found in the country with *An. funestus* s.s. being the most abundant species. Furthermore, *An. funestus* s.s. is the only one species in the group contributing to malaria transmission and have adapted biting times that allow them to bite at dawn. In addition, across the country, *An. funestus* were found resistant to pyrethroid insecticides used for bed nets impregnation and also resistant to bendiocarb which is currently being introduced in indoor residual spraying formulation in malaria endemic regions. All these findings highlight the challenges faced in controlling this malaria vector. Therefore, advancing the knowledge of vectorial competence of *An. funestus*, understanding the dynamics of insecticide resistance in this malaria vector, and exploring alternative vector control measures, are critical for sustainable malaria control efforts in Benin Republic.

**Supplementary Information:**

The online version contains supplementary material available at 10.1186/s12936-023-04815-9.

## Background

Malaria continues to burden communities living in endemic areas of Africa. In these countries, intense efforts are being made to bring malaria under control to move forward its elimination. In Benin, despite number of efforts to control malaria, the disease remains largely prevalent across the country [1]. Since 2011, the National Malaria Control Programme (NMCP) on behalf of the Ministry of Health, in collaboration with international organizations and partners, has implemented various malaria control strategies to reduce the burden of the disease [2]. Some of the key malaria control interventions in the country include mass distribution of insecticide-treated nets (ITNs) every 3 years, indoor residual spraying (IRS) and improved diagnosis and treatment by the deployment of anti-malarial drug-based interventions [2]. These efforts targeting either the parasites or vectors have resulted in a significant increase in the usage and ownership of impregnated bed nets, with rates reaching up to 85% [1, 3]. As results, this has contributed to a substantial reduction in malaria-induced morbidity and mortality [1]. Health authorities in Benin have also utilized behavioural change communication (BCC) strategies to raise awareness about malaria prevention and control measures, encouraging communities to adopt positive behaviours to protect themselves from malaria [4]. It has been recognized that vector control is the main malaria control measure that has significantly contributed to the decline in malaria burden through the use of ITNs and IRS [5] targeting mosquito vectors.

Among the malaria-transmitting vectors, *Anopheles funestus* is one of the primary species in sub-Saharan Africa, including Benin [6]. The *An. funestus* group comprises nine species distributed across sub-Saharan Africa including *An. funestus* sensu stricto (s.s.),* Anopheles vaneedeni*, *Anopheles leesoni*, *Anopheles parensis*, *Anopheles rivulorum*, *Anopheles fuscivenosus*, *Anopheles brucei, Anopheles aruni*, and *Anopheles confusus* [7, 8]. Only *An. funestus* s.s. is known to be involved in malaria transmission [7, 8]. All other species, except for *An. rivulorum*, appear not to be associated with a human host-seeking tendency. Indeed, some reports have demonstrated *An. rivulorum* contribution in malaria transmission [9, 10]. It is well known that effective vector control strategies heavily rely on better understanding the abundance dynamics of the malaria-transmitting vectors, such as *An. funestus*, and the factors influencing their ability to transmit the disease. Despite the significant implication of *An. funestus s.s.* in malaria transmission, few studies have been conducted in Benin on this malaria vector [11, 12]. The existing studies have primarily focused on investigating only its resistance status to the insecticides frequently used in public health [11, 12]. This highlights the necessity to gather comprehensive information which will significantly contribute to this vector control in Benin. Indeed, several research gaps remain in (i) understanding *An. funestus* vectorial competence (identification of transmission hotspots, influencing factors, vector-parasite interactions); (ii) advancing insecticide resistance monitoring and management (resistance mechanisms, its spread and dynamics); and (iii) identifying potential alternative vector control strategies (biological control, genetic control, integrated vector management).

So far in Benin, malaria vector control measures are regardless of individual members in *Anopheles* species. Therefore, controlling specifically *An. funestus* poses several challenges, such as (i) behavioural change in biting activity (females mosquitoes have a tendency to bite in the evening or before bedtime when people might not be protected by ITNs) [13, 14]; (ii) breeding habitat adaptability (larvae can adapt and thrive in various breeding habitats) [15, 16]; and (iii) insecticide resistance (*An. funestus* has shown a remarkable ability to develop resistance to commonly used insecticides, such as pyrethroids) [17, 18]. Addressing these challenges requires a multifaceted approach, including the development of new vector control strategies, and continued surveillance for insecticide resistance.

To help establish effective resistance management strategies, it is important to better understand the distribution of *An. funestus*, its insecticide resistance profiles with underlying mechanisms and its impact on effectiveness of control interventions and malaria transmission [19]. In this review, the primary aim was to comprehensively evaluate the prevailing distribution and vectorial competence of *An. funestus* in Benin. Additionally, the insecticide resistance patterns exhibited by this mosquito species are analysed and future perspectives for vector control strategies are discussed. This will provide valuable insights that can inform and the elaboration of evidence-based policy and the implementation of more effective vector control interventions in Benin Republic.

## Literature search

A literature search was performed using the search terms (*Anopheles funestus*; *An. funestus*; Benin Republic; insecticide resistance) with the Boolean operator (AND) as follow: “*Anopheles funestus* and Benin Republic”, “*An. funestus* and Benin Republic” and “Insecticide resistance in *An. funestus* and Benin Republic”. The search was limited to publications written in English and in French, and it was done using the most popular search platform Google, Google Scholar and PubMed databases. In addition, Google and Google Scholar automatically index most information from the academic web. All papers that reported collection of at least one *An. funestus* (based on morphological identification) and from the year 2007, where insecticide resistance monitoring in *An. funestus* in Benin Republic started, were considered. Finally, twenty-one (21) published studies reporting work undertaken between 2007 and 2019 in Benin were selected for this review.

## Distribution and density of *Anopheles funestus* populations

Most of the available data on *An. funestus* in Benin were recorded when conducting a survey on *Anopheles *gambiae sensu lato (s.l.). Since 2007. There has been a scarcity of entomological research available on *An. funestus* in Benin (Additional file 1).

**Fig. 1:**
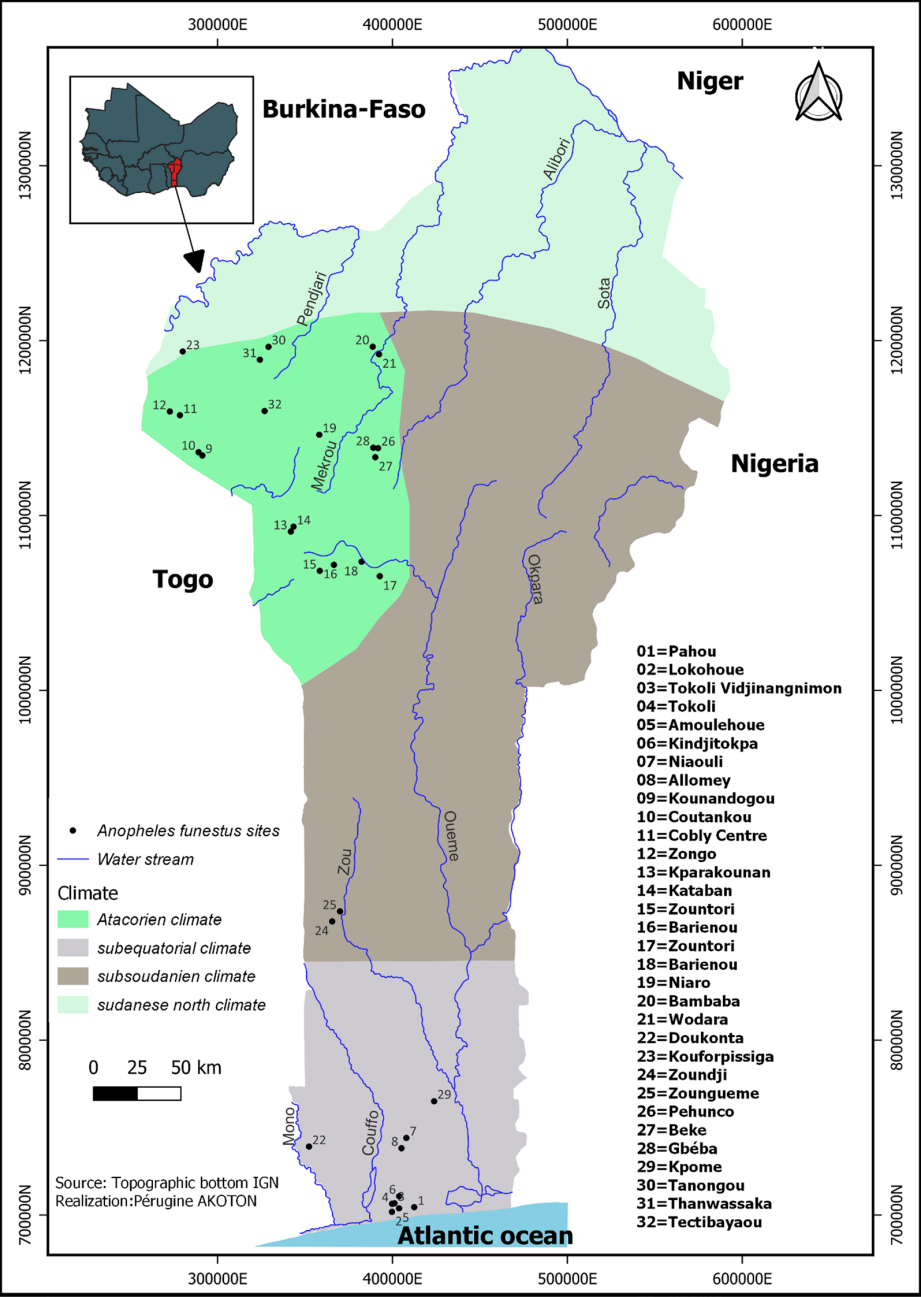
Distribution of *An. funestus* sites in Benin

Despite having a relatively lower population density compared to *An. gambiae* s.l., *An. funestus* is widely distributed in Benin. It has been found in the four climatic regions of the country, namely North Sudanese, wet Sudanese, Sub-Sudanese and Sub-equatorial as described by Djouaka et al. [20] (Fig. 1). Both North Sudanese and wet Sudanese climatic regions are characterised by a long dry and a short rainy season. Large water bodies are found in North Sudanese climatic region where temperatures are the highest particularly during the dry season [20]. However, the west part of the North Sudanese region is dominated by hills of up to 800 m of altitude and several small water bodies. The vegetation is partially made of wet savanna with the lowest temperature in the country, and rainfall ranging from 1200 to 1300 mm per year [20]. The sub-Sudanese climatic region covers the center of the country and part of the South. This region is less hilly and the vegetation is of wet savanna. Annual rainfall is between 900 and 1200 mm [15].

The sub-Equatorial climatic region covers the country’s southern part and extends up to the coastal areas. The relative humidity is high and can up to 95%, temperatures are relatively low ranging from 25 to 31 °C, and the vegetation is a mosaic of coastal wetlands, forests, and savannah [15].

*Anopheles funestus* was found in sympatry with *An. gambiae* and more often in the country’s western part and Savannah regions, as shown in Fig. 1. The relatively high occurrence of this species in the western regions of Benin could be attributed to the relatively low temperatures, humidity associated with the hilly landscape, and the presence of streams covered with vegetation [21].

In the eastern part, very little population of *An. funestus* was found, certainly due to the low rainfall and high dryness. The low occurrence of *An. funestus* population in this part of the country may also be attributed to the sampling period or the presence of a few permanent freshwater bodies covered with vegetation [22]. However, an extensive *An. funestus* surveys are required in this part of the country to confirm the observed lower occurrence.

During dry seasons within the study areas, *An. funestus* was found more abundant with a peak density during the dry-to-rainy transition period [20, 23]. This is probably due to their larval breeding habitats’ stability and adaptation to desiccation [24]. Moiroux and colleagues [25] have reported that in Ouidah-Kpomasse-Tori Bossito district located in Southern Benin, *An. funestus* becomes the predominant *Anopheles* mosquitoes during the dry-hot season. They get more aggressive during that period with a high density around localities bordering large water bodies such as the Toho Lake. Surveys carried out in Kpome (southern Benin) and Tanongou (northern Benin) showed a relatively high density of *An. funestus* (up to 3 mosquitoes/room) during the dry-to-rainy transition period [20]. High density of *An. funestus* was reported in other southern villages with 0.29 to 13.48 mosquitoes per village [6, 12]. The presence of *An. funestus* was also reported in others localities across most of departments in Benin, but at low density [23, 26–32]. Nevertheless, the increased *An. funestus* density in localities during the dry season contributes to the residual malaria transmission in Benin [20]. Indeed, during the dry season, mosquitoes’ aggressivity, especially malaria vectors, becomes very low [25]. In regards to this situation, vector control tools, such as ITNs are used less by inhabitants living in endemic regions and could impede the achievement of the World Health Organization (WHO) ultimate goal, which was to eliminate malaria in some countries by 2030 [33].

Furthermore, in Lokohoue and Tokoli villages located in OKT district (sub-Equatorial climatic regions), a human could receive 2.1 to 18.73 bites of *An. funestus* per night [34]. However, *An. funestus* was found to be relatively less aggressive in both rainy and dry seasons with 0.35 b/h/n (bite/human/night) and 0.84 b/h/n respectively in Copargo in wet-Sudanese climatic zone [23]. In Kérou and Djougou districts (wet-Sudanese climatic zone), a human could receive 0.66 and 6.55 bites of *An. funestus* mosquitoes per night, respectively [35, 36]. The lowest aggressivity observed in these regions may be due to the sampling period, rainy seasons (not much suitable for *An. funestus* development) and the rarity of stable larval habitats during the same period. Considering the information above and for a sustainable malaria control, it becomes important to redeploy vector control tools in the dry season because this season seems not to be a target period to the NMCPs for implementing these strategies.

## Contribution of *Anopheles funestus* to malaria transmission

Most members of *An. funestus* group are zoophilic (preference to take blood meals on animals), except *An. funestus* s.s. [37], which is a the primary vector for *Plasmodium* species [11, 20, 37–41]. Although, few studies have investigated the distribution of *An. funestus* members [11], PCR-based species identification of wild-caught *An. funestus* in various localities across Benin revealed two main sibling species, including *An. funestus* s.s. (the most abundant; up to 99%) and *An. leesoni* (less than 10%) [6, 20]. These two sibling species were mainly caught indoors confirming the endophilic (tendance to inhabit/rest indoors) behaviour of *An. funestus* circulating in Benin [40]. *Anopheles funestus*, when exhibiting indoor biting behaviour, increases the likelihood of direct contact with humans, typically during the night when they are not yet under the bed nets. This increases the risk of transmitting the malaria parasite. Furthermore, *An. funestus* may exhibit seasonal variations in biting behaviour, with an increased tendency to bite indoors during certain periods [13]. Such information can help to implement suitable vector control strategies during the times when transmission risk is high. Indoor biting behaviour of *An. funestus* should, therefore, be taking into account when developing novel strategies to control malaria vectors.

*Plasmodium* infection rate has been reported at a very high level in *An. funestus* s.s. in Benin [11, 20]. This rate ranged from 2.64 to 15.78% in mosquitoes from Pahou and Gakpé near the Toho Lake in OKT district in coastal area of Benin (Additional file 1). Also, a high *Plasmodium* infection rate has been reported in the southern inland region of Benin at Toffo and Lokossa with values of 13.33 to 18.51%, respectively [20].

Overall, high *Plasmodium* infection rates of *An. funestus* s.s. observed in some villages in the West part of Benin and its related high anthropophily (preference to take blood meals on human), confirm the role of this vector species in malaria transmission, exceeding in some cases, *An. gambiae* s.l. (Additional file 1). A relatively high *Plasmodium* infection rate of *An. funestus* s.s. collected indoors during the dry season was reported in Toffo district, where this specie competes with *Anopheles coluzzii*, one of the primary malaria vectors in southern Benin [11]. The infection rates recorded in both *An. funestus s.s.* and *An. coluzzii* during the same period may explain the high malaria transmission and incidence during the dry seasons in Toffo [11]. The same trend was observed in Copargo in the Donga department, where a high infection rate was also reported in *An. funestus*, despite the high abundance of *An. gambiae* s.l. [23]. The former (26.08%) exhibited three times higher sporozoite rate than the later (8.49%) [23]. In Tanongou neighborhood located in the Atacora department, *An. funestus s.s.* was also relatively highly infected with *Plasmodium* (5.62%) [20] (Additional file 1).

Considered as a main indicator to estimate the overall contribution of *Anopheles* mosquitoes to malaria transmission, the Entomological Inoculation Rate (EIR) is the product of sporozoite rate and human biting rate over a defined time and space [42, 43]. The EIR of both *An. gambiae* s.l. and *An. funestus* s.s. varied significantly according to the season. In fact, during the rainy season, 75 infective bites per person were attributed to *An. gambiae* s.l. while *An. funestus* s.s. ensured only 18.75 infective bites per human. In contrast, during the dry season, *An. funestus* s.s. accounted for 37.5 infective bites per human while *An. gambiae* s.s. resulted in 28.12 infective bites per human in Northern Benin [23]. A relatively similar infective bites per human per 100 nights has been reported in *An. funestus* s.s. (0.67 infective bites) and *An. gambiae* s.l. (1.38 infective bites) in southern Benin [6].

The ability of *An. leesoni* to transmit *Plasmodium* remains unexplored in Benin. None of the studies included in this review has described the presence of *Plasmodium* species in *An. leesoni*, suggesting that it may not be involved yet in malaria transmission in Benin [6, 20, 40] and highlighting that it has no public health significance in Benin [20]. In addition, *An. leesoni* has demonstrated a very high zoophilic behavior and has been found in sympatry with *An. funestus* s.s. in Tanguiéta, northwestern Benin [20]. Only one *An. leesoni* was found in the coastal area at Tokoli-Vidjinangnimon village in OKT district [6]. However, recent studies in Cameroon reported the presence of *An. leesoni* infected with *Plasmodium* spp. [38, 39] suggesting that, extended studies on *An. funestus* group in Benin might provide more valuable information regarding the implication of *An. leesoni* in malaria transmission in the country.

Other members of *An. funestus* group have not been found in Benin. However, certain members of this group have been reported to relatively contribute to malaria transmission in Nigeria and Tanzania [10, 40, 44]. Although, *An. rivulorum* was found with an anthropophilic rate (an important factor in vectorial capacity) of 40% in the southern region of Nigeria, its implication in malaria transmission in this country was not yet elucidated [40]. Further, specimen of *An. rivulorum* was positive for *P. falciparum* found in Tanzania [10, 44]. *Anopheles vaneedeni*, which can easily harbor *Plasmodium* parasites under laboratory conditions, is either exophilic or anthrophilic [9]. Another member of *An. funestus* group, *An. parensis*, was reported not susceptible to malaria parasite infection [40, 45, 46]. However, recent study reported 1.6% of *Plasmodium* infection rate in *An. parensis* in Mozambique [47].

Besides, the contribution of *An. funestus* to malaria transmission in the urban environment remains scanty in Benin while such studies in other African countries have shown the implication of *An. funestus* in malaria parasite transmission in the city of Yaoundé in Cameroon [38]. This instigates to further investigate the eco-epidemiological characteristics and behavioral traits of *An. funestus* mosquito in urban settings, in order to map malaria risk and burden and to improve current vector control strategies.

## Insecticide susceptibility profile

Until 2010, no data on insecticide susceptibility profile and mechanisms of insecticide resistance of *An. funestus* were published in Benin. From 2011, characterization of insecticide resistance in *An. funestus* populations have been reported [12]. Four class of insecticides were monitored: 4% DDT and 4% dieldrin (Organochlorines), 0.75% permethrin and 0.05% deltamethrin (Pyrethroids), 0.1% Bendiocarb (Carbamate) and 5% malathion (Organophosphate). *An. funestus* susceptibility to insecticides was assessed in only 5 locations across the country [11, 12, 20, 34, 48] (Additional file 2), indicating that there is a need to further investigate resistance status in this malaria vector to better characterize areas where high resistance levels prevail nationwide.

Pyrethroid resistance in this *An. funestus* seems to be spread across the country as its counterpart *An. *gambiae s.l. In addition, *An. funestus* collected in 2007 and 2008 was found to be fully susceptible to a diagnostic dose of deltamethrin in Tokoli and Lokohoué (Additional file 2) according to Moiroux et al. [34]. In contrast, *An. funestus* collected between 2009 and 2011 in Pahou near Tokoli and Lokohoué villages, were resistant to diagnostic doses of deltamethrin and permethrin with mortality rates of 66.4% and 88.8%, respectively [12]. Elsewhere, high resistance has been reported in *An. funestus* collected in 2014 in Kpome village, with mortalities rates of 13% and 46.5% for permethrin and deltamethrin respectively [11]. In addition, even when *An. funestus* from Kpome was exposed for 90 min, it was still resistant to diagnostic dose of permethrin (51.62% mortality) [11]. This constitutes a serious threat for the effectiveness of pyrethroid-based interventions.

In 2017, the same trend was observed in *An. funestus* from Kpome where resistance to diagnostic dose of permethrin and deltamethrin was recorded with mortality rates of 14.84% and 44.15%, respectively [48]. This resistance profiles suggest an increase in the overall level of pyrethroid resistance in southern Benin within 6 years (from 2011 to 2017) and might be attributed to the increased ITNs coverage across Benin and additional selection factors such as pesticides use in agriculture [49, 50].

Similarly, permethrin resistance was also observed in 2014 in Doukonta, located in Lokossa district in southern Benin with a mortality rate of 11% [20]. The presence of resistant *An. funestus* in inland areas of Kpome and Doukonta compared to coastal localities (e.g. Pahou) [20] may be associated with gene flow among *An. funestus* in south of Benin [11].

A comparative analysis of *An. funestus* collected from Doukonta and Tanongou located respectively in southern and northern transect of Benin, reveals contrasting resistance patterns in *An. funestus*, marked by a full susceptibility to permethrin in Tanongou compared to a resistance in Doukonta (Additional file 2).

In addition to the resistance to pyrethroids, *An. funestus* from Benin are also resistant to bendiocarb [11, 12]. This raises a concern for NMCPs because bendiocarb is currently being introduced in IRS formulation for malaria vector control in West African countries [51]. Indeed, bendiocarb resistance was observed in *An. gambiae* in Benin 3 years after the implementation of IRS [51] and such resistance may likely spread to *An. funestus* owing to its endophilic behavior.

A relative resistance to dieldrin was recorded in *An. funestus* mosquitoes from Pahou (mortality rate of 93%) while a susceptible status was observed in those from Kpome (mortality rate of 98.9%) [11, 12]. However, Both of them were resistant to DDT [11, 12, 20]. The recorded mortality after standard bioassay exposure ranged from 0 to 9.1% (Additional file 2). Meanwhile, a relative resistance was observed for DDT (mortality rate of 90 ± 3.18%) in Tanongou in northern Benin [20].

All *An. funestus* mosquitoes from southern Benin remain susceptible to malathion [11, 12]. Nevertheless, the eco-toxicity of this insecticide limits its adoption by national malaria vector control programmes. Overall, the multiple resistance in *An. funestus* species underline the complexity of malaria control in Benin.

Although, *An. funestus* became largely distributed in Benin and resistant to pyrethroid insecticides, it still remains less studied. Regular monitoring of insecticide susceptibility profiles of this malaria vector should be implemented to help NMCPs improve strategies made in place to control malaria.

## Mechanisms underlying the insecticide resistance phenotypes

A ratio of mosquito individuals can tolerate lethal doses of insecticides in a normal population of the same species through different mechanisms, such as (i) metabolic resistance: insecticide can be broken down or metabolized by detoxification enzymes much faster in the resistant mosquitoes than in the susceptible ones, thereby quickly eliminated from the mosquito organism; (ii) target-site resistance: the insecticide target-site can be modified due to the presence of mutation that prevent the insecticide from binding thereby reducing lethal effect of such insecticide; (iii) penetration resistance: resistant mosquitoes may limit penetration of the insecticides than susceptible insects, or (iv) behavioural resistance (the less studied): mosquitoes avoid the insecticide contact [52]. Characterizing resistance mechanisms is an essential step in insecticide resistance management. This provides baseline data for designing control programmes and evidence-based choice of insecticides.

Basically, biochemical and molecular analysis as well as synergistic tests are usually used to determine insecticide resistance mechanisms in *Anopheles* vectors exposed to insecticides. These analysis and synergistic assays were used to characterize the mechanisms involved in insecticide resistance. Whole genome data has also recently become available for *An. funestus* studies [53] and advance molecular researches on the insecticide resistance mechanisms developed by this vector was uncovered.

## Mechanisms underlying DDT, permethrin and deltamethrin resistance

A synergistic study conducted by Djouaka and collaborators [12], has suggested that P450 genes play very little role in the observed DDT resistance in *An. funestus* collected from Pahou (Southern Benin). Furthermore, authors showed that glutathione S-transferase (GSTe2) gene was overexpressed in these resistant *An. funestus* [12, 54]. Indeed, the *GSTe2* gene is 44.8 time overexpressed in DDT-resistant mosquitoes when compared to the susceptible strain (FANG) [54]. The *GSTe2* gene expression was higher than that of other GSTs genes in the same mosquito specimens indicating that *GSTe2* is likely the main detoxification gene associated with DDT resistance in *An. funestus* mosquitoes collected from Pahou [12, 54]. As a result, further studies are need to explore the genomic pathway conferring this selective advantage for GSTe2 gene.

Further, high frequency (96%) of the L119 F-*GSTe2* resistant allele in *An. funestus* collected from other localities of southern Benin was reported [11, 20]. However, a relatively lower frequency (35%) of this resistant allele was recorded in *An. funestus* collected from Tanongou (northern part of Benin) [20].

Interestingly a microarray-based genome-wide transcription and qRT-PCR analysis on *An. funestus* mosquitoes from Kpome (Southern Benin) showed that, overexpression of the *GSTe2* gene is responsible for the observed DDT resistance [54, 55]. It was also reported the consistent difference for this gene between the population of southern Benin (Kpome, Pahou and Doukonta) and that of Tanongou (North Benin) [55] indicating that possible barriers to gene flow exist between these populations. This implies that barriers to gene flow likely to impact the design and implementation of resistance management strategies in this country.

A number of studies have revealed that L119F-GSTe2 resistant allele is near fixation in both DDT susceptible and resistant *An. funestus* mosquito populations from Kpome, Pahou and Doukonta (Southern Benin) [11, 55, 56]. On the other hand, there is a possibility that the L119F-GSTe2 allele could also be selected by pyrethroid-based interventions [11].

Although resistance genes confer the potential of surviving insecticide exposures, they are often associated with pleiotropic effects on various fitness-related traits in *An. gambiae* mosquitoes (e.g., trophic behaviour, fecundity, fertility, parasite transmission, longevity, and larval survivorship) [57, 58]. Influence of GST-metabolic resistance on vectorial competence in *An. funestus* should be more investigated.

Resistance to permethrin and deltamethrin in *An. funestus* across southern Benin was mainly attributed to cytochrome P450 monooxygenases [48, 56]. For instance, a significant over-expression of two duplicated P450s, *CYP6P9a* (Fold Change: 4.7) and *CYP6P9b* (Fold Change: 7) which can metabolize both permethrin and deltamethrin [59] was observed in *An. funestus* from Pahou (Southern Benin) [12]. Overall, there is a need to continue characterizing the insecticide resistance mechanisms in all localities where the presence of *An. funestus* have been reported in Benin to further capture the spread of this vector resistance and underlying mechanisms. This could help Benin’s NMCP to design and implement more suitable measures for resistance management in malaria vectors.

## Mechanisms underlying bendiocarb and dieldrin resistance

Although, *An. funestus* populations from Pahou and Kpome were resistant to bendiocarb with a mortality rate ranging from 64 to 70%, no evidence of *ace-1R* resistant allele was recorded in this carbamate-resistant populations [11, 12]. A resistance profile of *An. funestus* to bendiocarb should be further investigated to provide updated information important in improving current vector control strategies in the country. In contrast, as resistance to dieldrin was recorded in Pahou, with susceptibility to the same insecticides in Kpome were recoded; pyrosequencing and PCR-RFLP analysis performed on mosquito specimens from Pahou, revealed a moderate level (16%) of 296 S-Rdlr mutation, a GABA receptor mutation conferring dieldrin resistance in *An. funestus* in Africa [60]. Genotyping of the A296S-Rdlr mutation in *An. funestus* from Kpome revealed 99% homozygous susceptible genotype with 1% heterozygous further confirming susceptibility profile to this insecticide [11]. To date, no other study has been conducted to update dieldrin susceptibility profile in *An. funestus*.

## Perspectives on vector control strategies

A number of entomological, epidemiological and genomic studies on *An. gambiae* have significantly contributed to strengthen malaria control. The control of the major malaria vectors in endemics area is one of an important ways to interrupt the transmission of this disease [61]. Therefore, phase I (Cone and Tunnel tests) and Phase II (Experimental Hut Trials, EHTs) extend evaluation studies are also needed to appreciate the effectiveness of current and new designed vector control products targeting *An. funestus* mosquitoes.

In Benin, little is known about *An. funestus* responses to vector control tools. Bio-efficacy assays throughout standard cone and tunnel tests showed the loss of efficacy of currently used ITNs (PermaNet 2.0 and Olyset Net) against natural population of *An. funestus* from Kpome [48].

EHTs were also conducted in Kpome where more than 40% and 60% of *An. funestus* survived in presence of PermaNet 2.0 and Olyset Nets, indicating that these nets were able to provide only 46% and 17% personal protection, respectively [48]. Furthermore, it has been reported that current ITNs were still estimated to provide average ‘true’ personal protection of 80% against *An. funestus* bites in Lokohoue village in Ouidah in southern Benin [62]. All of these findings suggest that protective effect of currently used nets is compromised in Benin. Surprisingly, it was observed in Lokohoue that *An. funestus* likely continues to bite at dawn when people are no longer sleeping under mosquito nets [62]. This is a huge concern since current vector control strategies, rely only on the use of pyrethroid-treated nets to target nocturnal, endophilic malaria vectors.

However, the fact that *An. funestus* is still susceptible to bendiocarb in southern Benin and considering its high endophilic and endophagic behaviors, IRS in combination with current ITNs may be a promising control strategy, especially during the dry season where the density of *An. funestus* is high. Also, the major role played by metabolic resistance to pyrethroids in *An. funestus* in Benin suggests that combining the synergist PBO, such as PBO-Pyrethroid bednets, could help manage the pyrethroid resistance in this malaria vector. Interestingly, EHTs showed a benefit to use both PermaNet 3.0 and Olyset Plus (PBO-Based nets) to control resistant population of *An. funestus* in southern Benin [48]. A combination of the synergist PBO to pyrethroids makes treated-nets more efficient, as PBO is a potent cytochrome P450s enzyme inhibitor. Indeed, PermaNet 3.0 and Olyset Plus, were able to prevent blood feeding in 92% and 100% in resistant *An. funestus*, respectively [48]. High killing effect was also exhibited by these nets (100% for PermaNet 3.0 and 87% for Olyset Plus) [48]. The same trend have been observed for *An. gambiae* with 80% of personal protection in presence of PermaNet 3.0 [48].

Therefore, a combined pyrethroids-PBO nets showed a greater efficacy against resistant malaria vector populations and could be a promising strategy against pyrethroid-resistant *Anopheles* mosquitoes [63–67]. However, its efficacy can be impacted by other resistance mechanisms in mosquito vectors, such as GST-mediated metabolic resistance, which is not affected by PBO synergistic action [68]. Moreover, recent study revealed efficacy-loss of PBO–based nets (Olyset Plus) in highly pyrethroid-resistant *An. funestus* populations from Mozambique [69] and Cameroon [70]. This could also be occurring in Beninese mosquito populations. In fact, this reduced efficacy of PBO-based tools could be attributed to the overexpression of the cytochrome P450 genes that could allow mosquitoes to tolerate exposure to ITNs impregnated with pyrethroids and PBO in the nets. Efficacy against *An. funestus* of other new generation nets could also be explored as the case of *An. gambiae* where an interesting information have been reported in Benin and Ivory coast, highlights that these new generation nets have the potential to improve malaria vector control and provide better community protection against clinical malaria in pyrethroid resistant areas compared to standard pyrethroid-only ITNs [71–73].

The new generation nets combining pyrethroid and insect growth regulator (Pyriproxyfen) and a Royal Guard or Interceptor G2 impregnated with Chlorfenapyr (a pyrrole insecticide class), have shown a great efficacy against resistant *An. gambiae* in West African countries [71–73]. More recently, it was reported that Interceptor G2 provided a high lethal effect, blood-feeding inhibition, repellency and personal protection against *An. funestus* from Tanzania [74]. These new bednets could be a potential insecticide resistance management tool to prevent malaria transmission in areas compromised by the spread of pyrethroid resistance. Additionally, the insect growth factor combined with pyrethroid may also positively impact the reproductive success in *An. gambiae* [73]. Further investigations on how *An. funestus* exposure to these new nets can affect the fecundity, fertility and other life history traits, could provide additional key information on fitness effects.

Definitely, more robust entomological studies focusing on *An. funestus* should be implemented in Benin. Indeed, in addition to existing assays to measure the performance of ITNs which are rely on detection of rapid knockdown and 24-hour mortality, critical insights into the behavior of mosquitoes in response to ITNs can be gained by laboratory and semi-field studies that quantify important traits including net contact time, and blood-feeding behaviour, longevity and reproductive features. A suite of experimental procedures ranging from simple benchtop assays (e.g.Video Cone test, Thumb/Baited Box test) to large-scale video tracking [75, 76] to record the lifetime impact of exposure to an active ingredient in presence of a host could provide robust parameters to better appreciate the effectiveness of the current and new generation vector control tools.

On the other hand, alternative strategies using non-insecticide-based tools in combination with current ITNs against resistant pyrethroid resistant mosquitoes could be explored to improve progress towards malaria elimination. New tool in development uses combination of human-associated stimuli, including olfactory, visual and thermal cues, to lure and kill malaria vectors without insecticides. The strategy could be a promising integrated vector control strategy and have a real public health benefits.

In addition, considering the insecticide resistance phenomenon, novel innovative symbiotic control measure may be also explored [77, 78]. However, before implementing this control measure in natural *An. funestus* mosquito populations, it will be useful to better understand the bacterial diversity in this vector and their interactions with their hosts. Indeed, one of key factors determining vector competence is the gut microbiota of the mosquito [79]. The microbiota in mosquito midguts plays a crucial role in the development, reproduction, immunity, and vector competence as reviewed in *An. gambiae* populations [80, 81]. Recently, several research reports showed associations between the mosquito microbiota and resistance to the current insecticides used for vector control [82–84]. A good understanding of the role of *An. funestus* microbiota in insecticide resistance will allow improvement of techniques toward curbing the widespread of insecticide resistance in malaria vector [85].

## Conclusion

This review provides synthesized information on the vectorial competence of *An. funestus*. *Anopheles funestus* became largely distributed in Benin with high density recorded during the driest period. The prevalence of *Plasmodium* infection in *An. funestus* was high with comparable entomological inoculation rate between this species and its counterpart *An. gambiae*. Furthermore, *An. funestus* was found to be resistant to permethrin, deltamethrin and bendiocarb, resistant to DDT but remain susceptible to malathion. GSte2 and P450 genes are mainly incriminated in the observed phenotypic resistance, highlighting the urgent need for further actions to strengthen malaria control strategies. Information provided on mechanisms underlying insecticide in *An. funestus* call for the development of more comprehensive resistance management and the implementation of alternative control interventions.

### Supplementary Information

** Additional file 1.** Overall *Plasmodium* infection rates in *Anopheles* mosquito populations from Benin.

** Additional file 2.** Evolution of insecticide resistance in *An. funestus* populations from Benin.

## Data Availability

All data generated or analysed during this study are included in this published article and additional files.

## Abbreviations

DDT: Dichlorodiphenyltrichloroethane
GABA: Gamma-aminobutyric acid
GST: Glutathione-*S* transferase
IRS: Indoor residual spraying
ITNs: Insecticide-Treated Nets
PBO: Piperonyl butoxide
qPCR: Quantitative polymerase chain reaction
RFLP: Restriction fragment length polymorphism
EHT: Experimental hut trials
