# Supplementary material for: Vectorial competence, insecticide resistance in Anopheles funestus and operational implications for malaria vector control strategies in Benin Republic
Source: Malar J. 2023 Dec 21;22:385. doi: 10.1186/s12936-023-04815-9 (PMC10740250; doi:10.1186/s12936-023-04815-9)
Supplement: Supplementary file 1 — Additional file 1. Overall Plasmodium infection rates in Anopheles mosquito populations from Benin. [file 12936_2023_4815_MOESM1_ESM.docx]

**Additional file 1.** Overall *Plasmodium* infection rates in *Anopheles* mosquito populations from Benin

| **Villages** | **Municipality** | **Collection periode** | **Season** | **Species** | **Number of collected *Anopheles*** | ***Plasmodium* infection (%)** | **Ref** |
| --- | --- | --- | --- | --- | --- | --- | --- |
| Lokohoue, Tokoli Vidjinangnimon, Amoulehoue and Kindjitokpa | Ouidah | October 2007 to May 2008 | RS and DS | *An. funestus s.s*.  *An. leesoni* | 577 | 2.6 | [6] |
|  |  |  |  |  | 1 |  |  |
|  |  |  | RS | *An. Coluzzii* | 101 | 5.94 |  |
|  |  |  | DR | *An. gambiae* | 217 | 11.52 |  |
| Pahou | Ouidah | July 2009 to  April 2011 | RS | *An. funestus s.s.* | 300 | Not available | [12] |
|  |  |  | DS |  |  |  |  |
| Tokoli and lokohoue | Ouidah | November 2007 and March 2008 | DS | *An. funestus s.l.* | 381 | 2.62 | [25] |
|  |  |  | DS | *An. gambiae s.s.* | 94 | 5.31 |  |
|  |  | October 2007 to  May 2008; November 2008 to June 2009; and April 2011 | DS and RS | *An. funestus s.l.* | 1866 | Not available | [34] |
|  |  |  | DS+RS | *An. gambiae s.s.* | 367 |  |  |
| Gbéba, Niaro, Zongo, Coutankou, Kounandogou,Thanwassaka and Tectibayaou | Pehunco; Kouandé; Cobly; Boumkoumbe; Tanguieta; Toucoutounan | September to October 2010 | RS | *An. funestus s.l.* | 13 | Not available | [26] |
| Allomey and Niaouli | Allada | March to November 2012 | RS | *An. funestus s.l.* | 18 | Not available | [28] |
| Kpome | Toffo | December 2013 to February 2014 | DS | *An. funestus s.s.* | 104 | 18.27 | [11] |
|  |  |  | DS | *An. coluzzii* | 93 | 13 |  |

| Pahou, Kpome, Doukonta, Zoundji, Zoungueme, Cobly centre, Kouforpissiga and Tanongou | Ouidah; Toffo; Lokossa; Savalou; Tanguieta; Materi and Cobly | December 2014 to January 2016 | DS and RS | *An. funestus s.s.* | 501  51 | 0 to 18.51 | [20] |
| --- | --- | --- | --- | --- | --- | --- | --- |
|  |  |  |  | *An. leesoni* |  | 0 |  |
| Zakekere | Cove | February 2009 to January 2010 | RS and DS | *An. funestus s.s.* | 21 | 0 | [27] |
|  |  |  | RS and DS | *An. coluzzii* | 8285 | 4.17 |  |
| Zountori and Barienou | Djougou | May 2016 to February 2017 | RS | *An. funestus s.l.* | 60 | Not available | [29,36] |
|  |  |  |  | *An. gambiae s.l.* | 1086 |  |  |
| Kataban and Kparakounan | Copargo | January to December 2013 | RS | *An. funestus s.s.* | 34 | 29.4 | [23] |
|  |  |  | DS |  | 81 | 24.7 |  |
|  |  |  | RS | *An. gambiae*  *An. coluzzii* | 455 | 8.8 |  |
|  |  |  | DR |  | 193 | 7.8 |  |
| Makrou-Gourou and Niarosson | Kouande | January to December 2016 | RS and DS | *An. funestus s.s.* | 40 | 12.5 | [41] |
|  |  |  |  | *An. gambiae s.l.* | 231 | 16.88 |  |
| Bambaba, Wodara, Pehunco and Beke | Kerou and Pehunco | September to October 2017 | RS | *An. funestus s.l.* | 22 | Not available | [35] |
|  |  |  |  | *An. gambiae s.l.* | 966 |  |  |

**RS**: Rainy Season; **DS**: Dry Season; **Ref**: References
