# Supplementary material for: Vectorial competence, insecticide resistance in Anopheles funestus and operational implications for malaria vector control strategies in Benin Republic
Source: Malar J. 2023 Dec 21;22:385. doi: 10.1186/s12936-023-04815-9 (PMC10740250; doi:10.1186/s12936-023-04815-9)
Supplement: Supplementary file 2 — Additional file 2. Evolution of insecticide resistance in An. funestus populations from Benin. [file 12936_2023_4815_MOESM2_ESM.docx]

**Additional file 2.** Evolution of Insecticide resistance in *An. funestus* populations from Benin

| **Location (municipality)** | **Climatic zone** | **Study year** | **Insecticide family** | **Insecticide** | **Mortality rate (%)** | **Resistance status** | **Ref** |
| --- | --- | --- | --- | --- | --- | --- | --- |
| Tokoli and Lokohoue (Ouidah) | Coastal region (Sub-equatorial) | 2007 and 2008 | Pyrethroid | Deltamethrin | 100 | Full susceptible | [34] |
| Pahou (Ouidah) | Coastal region (Sub-equatorial) | 2009 and 2011 | Pyrethroid | Permethrin | 66.4 | Resistance | [12] |
|  |  |  |  | Deltamethrin | 88.6 | Resistance |  |
|  |  |  | Organochlorine | DDT | 0 | High Resistance |  |
|  |  |  |  | Dieldrin | 93.3 | Moderate resistance |  |
|  |  |  | Carbamates | Bendiocarb | 64.5 | Resistance |  |
|  |  |  | Organophosphate | Malathion | 100 | Full susceptible |  |
| Kpome (Toffo) | Inland region / Sub-equatorial | 2014 | Pyrethroid | Permethrin | 13 | High Resistance | [11] |
|  |  |  |  | Deltamethrin | 46.5 | Resistance |  |
|  |  |  | Organochlorine | DDT | 9.1 | High Resistance |  |
|  |  |  |  | Dieldrin | 98.9 | Susceptible |  |
|  |  |  | Carbamates | Bendiocarb | 69.8 | Resistance |  |
|  |  |  | Organophosphate | Malathion | 100 | Full susceptible |  |
|  |  | 2017 | Pyrethroid | Permethrin | 14.8 | High Resistance | [48] |
|  |  |  |  | Deltamethrin | 44.1 | Resistance |  |
| Doukonta (Lokossa) | Sub-equatorial | 2014 to 2016 | Pyrethroid | Permethrin | 11 | High Resistance | [20] |
|  |  |  | Organochlorine | DDT | 8 | High Resistance |  |
| Tanongou (Tanguieta) | Wet Sudanese climatic region | 2014 to 2016 | Pyrethroid | Permethrin | 100 | Full susceptible |  |
|  |  |  | Organochlorine | DDT | 90 | Resistance |  |

**DDT** : Dichlorodiphenyltrichloroethane; **Ref** : References
